# Changes in physical activity, diet, and body weight across the education and employment transitions of early adulthood: A systematic review and meta‐analysis

**DOI:** 10.1111/obr.12962

**Published:** 2020-01-19

**Authors:** Eleanor M. Winpenny, Miranda Smith, Tarra Penney, Campbell Foubister, Justin M. Guagliano, Rebecca Love, Chloe Clifford Astbury, Esther M.F. van Sluijs, Kirsten Corder

**Affiliations:** ^1^ UKCRC Centre for Diet and Activity Research (CEDAR) at the MRC Epidemiology Unit University of Cambridge School of Clinical Medicine, Box 285, Institute of Metabolic Science, Cambridge Biomedical Campus Cambridge UK; ^2^ Newnham College University of Cambridge Cambridge UK

**Keywords:** obesity, school, university, work

## Abstract

Early adulthood is a time when individuals go through important life transitions, such as moving from high school into higher education or employment, but the impact of these life transitions on changes in body weight, diet, and physical activity is not known. We searched six electronic databases to July 2019 for longitudinal observational studies providing data on adiposity, diet, and/or physical activity across education or employment transitions in young people aged between 15 and 35 years. We found 19 studies, of which 17 assessed changes in physical activity, three body weight, and five diet or eating behaviours. Meta‐analysis (n=9) found that leaving high school was associated with a decrease of −7.04 (95% CI, −11.26, −2.82) min/day of moderate‐to‐vigorous physical activity. Three studies reported increases in body weight on leaving high school. A small number of studies suggested decreases in diet quality on leaving high school (n=2/4 papers) and leaving university (n=1) but not on starting employment (n=1). Studies suggested no change in physical activity on leaving university (n=4) but decreases in physical activity on starting employment (n=2/3). The transition of leaving high school is an important time to support individuals to prevent decreases in physical activity and gains in body weight.

## 1 INTRODUCTION

The period of life from adolescence to early adulthood has been suggested as an important developmental period for the establishment of long‐term patterns of obesity‐related lifestyle behaviours.^1^ This is a period of rapid psychological development,^2^ characterized by increasing personal autonomy and transitions between different social roles.^3^ In addition, individuals experience major life transitions during the early adulthood period, which may disrupt existing habits and allow new behaviour patterns to form.^4^, ^5^ These transitions include changes in the home environment, with individuals often moving out of the family home to live independently, changes in the school/work environment from high school to further education and into employment, and changes in social environments, with individuals moving from family dependence towards stronger peer networks and the development of partner relationships.

The period from adolescence into early adulthood (ages 15 to 35 years) is the age range in which prevalence of overweight and obesity rises the fastest.^6^ During this period, physical activity decreases,^7^ and changes in diet are observed, including decreases in fruit and vegetable consumption in adolescence and increases in diet quality in early adulthood.^8^, ^9^ Lifestyle behaviours developed during early adulthood are thought to track into adulthood, influencing later health outcomes.^10^ Study of this transitionary period presents an opportunity to understand the impact of changing social and physical environments on lifestyle behaviours, in order to support the development and targeting of public health policy and interventions.

There has been considerable interest in recent years in the “Freshman 15″ phenomenon: the popular belief that students gain 15 lb (6.8 kg) of weight in their first year of college in the United States. Longitudinal cohort studies across several countries have provided evidence to support this observation, although typically to a smaller extent, recently estimated at 7.5 lb (3.38 kg).^11^, ^12^ However, the transition to university is only one of several education and employment transitions that take place during early adulthood and only applies to the segment of the population who attend university. More universal life transitions during early adulthood include leaving high school and starting employment. There is some evidence that transitions between different education and employment states during this period contribute to changes in lifestyle behaviours (eg, diet^13^ and PA^13^, ^14^, ^15^) and may in part be driving the substantial weight gain observed in early adulthood.^14^, ^15^

In this study, we aimed to systematically review the existing literature on changes in adiposity and related lifestyle behaviours (diet, eating behaviours, and physical activity) across common education and employment transitions in late adolescence and early adulthood (15 to 35 years). We address the research question: how are changes in adiposity, physical activity, diet and eating behaviours associated with early adulthood education or employment transitions?

## 2 METHODS

The reporting of this study was guided by the Preferred Reporting Items for Systematic Reviews and Meta‐Analyses.^16^ Details of the protocol for this systematic review were registered on PROSPERO (ref: CRD42018106943) and can be accessed at https://www.crd.york.ac.uk/PROSPERO/display_record.php?RecordID=106943.

### 2.1 Information sources and literature search

This review was conducted as part of a suite of reviews examining the impact of early adulthood life transitions on changes in adiposity, diet, and physical activity. We conducted a systematic literature search of longitudinal observational studies providing data on adiposity, diet, and/or physical activity across life transitions in young people aged between 15 and 35 years, searching six electronic databases (MEDLINE, Embase, PsycINFO, Scopus, ASSIA, and Web of Science). The original search was conducted in July 2018 and updated in July 2019. The search strategy was built around three themes: outcomes (adiposity, physical activity, diet, and eating behaviours), life transitions (including entering or leaving education and employment), and study type (longitudinal, prospective). The full list of search terms is available in Table S1. Since the search was undertaken as part of a broader literature search considering additional transitions (eg, cohabitation and parenthood), the search strategy also contains terms relevant to other transitions.

### 2.2 Eligibility criteria

Inclusion and exclusion criteria for this review are presented in Table 1. Inclusion was restricted to those studies which report change in adiposity, diet, eating behaviours, or physical activity across an education or employment transition. We defined education and employment transitions to include entering or leaving education and entering or leaving employment. We included studies which reported longitudinal data with at least two data collection points, including one before and one after a transition, and within the specified age range (15‐35 years). We included adolescents as young as 15 years to capture transitions occurring from mid‐adolescence to early‐adulthood and limited the age range to 35 years, which has been proposed as the end of young adulthood with regards to weight control^17^. All articles published in the English language in a scientific journal were considered for inclusion, regardless of country of origin.

**Table 1:** Inclusion criteria

	Inclusion Criteria	Exclusion Criteria
Setting	All countries	None
Participants	Those aged between 15 and 35 years, inclusive (at least 2 time‐points within that range)	Those aged below 15 years of age or above 35 years of age Participant groups selected based on a preexisting health condition (including prediabetes but excluding weight status)
Exposure	Life transitions related to entering or leaving education and employment: eg, leaving high school, starting postsecondary education, leaving post‐secondary education, starting employment, and leaving employment	Papers that do not include data pre and post an education or employment transition
Outcomes	Individual level change in the following outcomes: Diet: intake of energy, macronutrients, foods, food groups, and dietary patterns Eating behaviours: eg, eating outside the home, fast food consumption, social aspects of eating, meal and snack consumption, and cooking Physical activity: moderate‐to‐vigorous physical activity, vigorous physical activity, light physical activity, total activity, sport participation, active travel, and energy expenditure Adiposity: BMI, body weight, body composition, anthropometry, overweight, and obesity	Studies including no dietary, PA, or adiposity outcomes Studies reporting tracking of outcomes only with no data on absolute change in behaviour Studies reporting solely on alcohol intake Studies reporting on eating disorders or weight reduction behaviour Studies reporting on dietary supplements only
Study type	Longitudinal prospective quantitative studies, with data reported including on specified outcomes before and after becoming a parent Observational analyses of longitudinal data that was originally collected as part of an intervention/trial but reported as a separate analysis	Other quantitative study types Qualitative study Intervention studies/trial analyses Reviews Case‐control studies Retrospective study
Publication type	Journal article	Conference abstract, study protocol, report, dissertation, book and professional journal
Publication year	Any	
Language	English	All other languages

### 2.3 Study selection

Titles and abstracts of each paper were first screened by one of five reviewers. In order to agree on a consistent approach between the reviewers, the screening process was first piloted. Two sets of three reviewers independently reviewed three sets of 500 titles/abstracts. Iterative comparison and discussion within the group allowed for development of a consistent screening approach, which was then applied to the remaining titles/abstracts. Those references that passed the title/abstract screening were then independently screened by two reviewers for inclusion, and any discrepancies were discussed with a third reviewer to reach consensus. Following full‐text screening, the included references were divided according to the transition(s) included and assigned to different reviews. Hand searching of the reference lists of publications included in this review identified three additional full texts to be considered for inclusion, none of which were included after full text screening.

Where data on the same study, transition(s) and outcome(s) were reported in multiple papers we selected one paper to be included in the analysis, using the following decision hierarchy: (a) papers where continuous outcome data were available and (b) data included separately for men and women.

### 2.4 Data extraction

Data were extracted from each included paper by one reviewer and checked by a second. Data extracted from each paper included study (cohort) name, country of cohort, ethnicity, sex and socio‐economic status of participants, details of the population from which the sample were recruited, and details on the transitions and outcomes reported, year of baseline data collection, and time between waves of data collection. Further details were extracted for each wave of data collection: sample size, mean age of the cohort, and mean and standard deviation (SD) of each relevant outcome. Where population subgroups where reported, eg, by sex or groups going through different transitions, outcomes were extracted for each subgroup.

### 2.5 Quality assessment

Included papers were assessed for methodological quality using a 10‐item quality assessment scale (see Table S2). This scale was created by combining items from a suite of scales, which have been specifically designed for longitudinal observational studies of adiposity, physical activity, and diet,^18^, ^19^ based on the quality criteria list for observational longitudinal studies of Tooth et al.^20^ Multiple papers from the same study were assessed separately because they may contribute independently to the analysis of different outcomes. The scale assessed four dimensions of methodological quality: (a) study population and participation rate, (b) study attrition, (c) data collection, and (d) data analysis. Two reviewers independently assessed each paper, with discussion to resolve any disagreements. The total score was reported for each paper to provide an indication of the overall quality of the paper. A score of 7 or more (out of 10) was judged to represent high methodological quality.

### 2.6 Data preparation

Papers were first categorized according to the transition and outcomes reported. Based on the numbers of papers available addressing the different exposures and outcomes, papers which reported changes in PA across the transition of leaving high school were assessed for inclusion in a meta‐analysis. Unadjusted measures of PA, reported before and after the transition, or change in PA across a transition, were converted to a standard metric of moderate‐to‐vigorous physical activity (MVPA) in minutes per day (min/day). Where continuous PA data were reported, but not in an appropriate format, ie, graphically, or without standard errors reported (two papers),^21^, ^22^ we contacted the authors to request further information. For conversion from MET‐minutes to MVPA we divided by 5, assuming a MET‐value of 5 for MVPA, in line with a previous approach.^23^ Where participation in MVPA was reported as number of times per week, we assumed 30 minutes of activity per occasion.^24^ With the exception of one study which reported changes in MVPA and associated SD across the transition,^25^ we calculated change in MVPA and the standard errors from reported pretransition and posttransition measures. Only one study reported data on the correlation between repeated measures within individuals, and this study was not representative of the remaining included studies in terms of methods and data quality. Therefore, we assumed a within‐person correlation of 0.5, applying the methods set out in the Cochrane Handbook (Section 16.1.3) to impute SDs and thus standard errors.^26^ As sensitivity analyses we also tested correlations of 0.4 and 0.6, as well as variation in correlations between studies; however, these changes did not substantially alter the main findings.

### 2.7 Synthesis of results

The included papers were heterogeneous, reporting on a number of different education and employment transitions (exposures) and diet, physical activity, or weight outcomes. We synthesized results across each of the different combinations of exposure and outcome reported. Where more than five studies were available reporting on the same combination of exposure and outcome (ie, leaving high school and changes in physical activity), we conducted a meta‐analysis to combine data across these studies (Stata version 15, StataCorp, USA). Random effects meta‐analysis was used, to account for differences between studies, using the method of DerSimonian and Laird,^27^ with the estimate of heterogeneity being taken from the inverse‐variance fixed‐effect model. The heterogeneity of associations was expressed using the *I* 2 statistic. Funnel plot asymmetry and Eggers test for bias were conducted for all meta‐analyses to investigate various forms of potential publication bias.

Fewer than 10 studies were available for any exposure and outcome combination, so we did not conduct meta‐regression to further understand the heterogeneity in our data, in line with guidance from the Cochrane Collaboration.^26^ Instead, we performed two separate subgroup analyses. We performed stratified meta‐analysis by sex, based on previous evidence that decreases in physical activity across early adulthood are greater in males than females.^7^ We also performed a stratified meta‐analysis across different categories of transition (ie, leaving high school and starting university vs leaving high school to all destinations).

## 3 RESULTS

**Figure 1:**
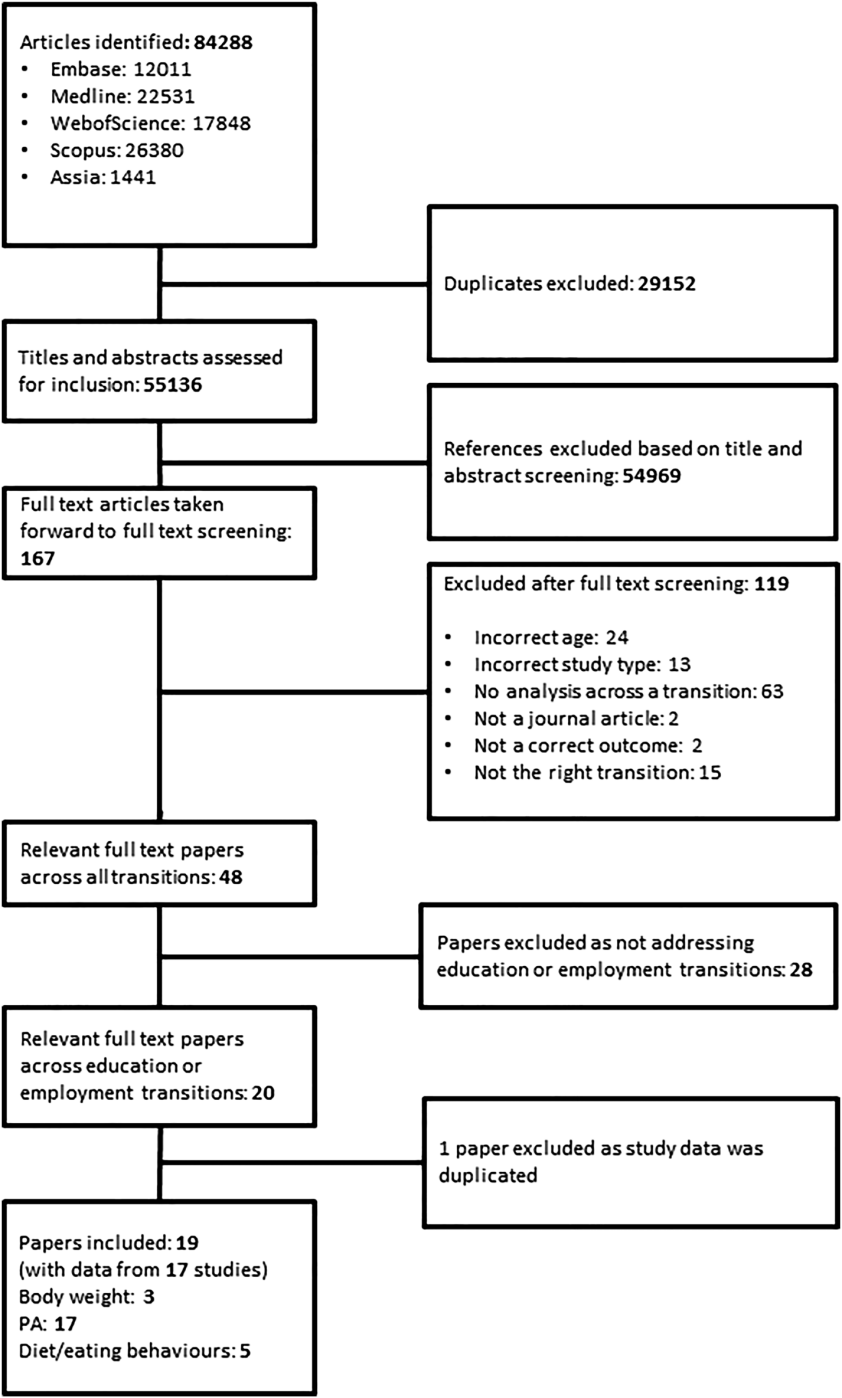
PRISMA flow diagram depicting study search, screening and inclusion process

Literature searches across the suite of reviews identified 55,136 titles and abstracts after removal of duplicate references, which were assessed for inclusion; 54,969 papers were excluded based on title/abstract screening. Of the remaining 167 papers taken forward to full text screening, 119 were excluded for the reasons shown in Figure 1. The 48 full text papers remaining were split by life transition with 20 identified as investigating education or employment transitions. Of these, 1 paper was excluded as it only included duplicate data also available in another included paper.^28^

A summary of results across the education and employment transitions included in this review is shown in Table 2, with further details discussed under each heading below. Study characteristics for the 19 papers included in this review are reported in Table 3. All the included papers reported on data from high‐income countries, with the majority of studies taking place in the United States (n=8 papers) or Australia (n=5 papers). Most papers (n=12) reported on data collected within the last 20 years, with the oldest study reporting on baseline data collected in 1991.^9^

**Table 2:** Summary table of findings across outcomes and education and employment transitions

	Body Weight	Physical Activity	Diet
Leaving high school and/or starting university	3 studies Increase (3/3 papers)	12 studies Decrease (7/12) Meta‐analysis (n=9): −7 min/day of MVPA	4 studies Mixed findings Decrease in fruit and dairy consumption (2/4)
Leaving university	0 studies	4 studies No change (4/4)	1 study Increase in confectionery and sugar‐sweetened beverage consumption (1/1)
Starting employment	0 studies	3 studies Decrease (2/3)	1 study No change (1/1)

Numbers in brackets indicate the number of papers, out of the total number that addressed that question, that report the direction of change stated.

**Table 3:** Descriptive characteristics of included papers

Paper	Study Name	Country	Baseline Year	N in Analysis Sample	% Female	Baseline Age (y), Mean (SD)^a^	Time to Follow‐up (y)	Transition	Outcome	Meta‐analysed	Quality Assessment Score
Baum, C. L. (2017)^34^	NLSY97	US	1998‐2008	2352	46	18‐19	1	Leaving high schoolStarting university	Body weight	N	3
Bell, S. and C. Lee (2005)^38^	ALSWH	Australia	1996	8545	100	20.7 (1.48)	4	Starting employment	PA	N	7
Brown, W. J., et al. (2009)^39^	ALSWH	Australia	2000	7173	100	22‐27	3	Starting employment	PA	N	6
Carney, C., et al. (2000)^37^	N/R	Scotland	1996	388	48	24.5 (3.6)	0.5	Leaving university	PA	N	7
Deforche, B., et al. (2015)^13^	N/R	Belgium	2008 & 2009	291	67	17.2 (0.5)	1.5	High school to university	Body weight, PA, Diet	Y (U)	9
Eime, R. M., et al. (2016)^22^	N/R	Australia	2008	84	100	16.2 (0.6)	1	Leaving high school	PA	Y (A)	6
Hootman, K. C., et al. (2018)^29^	N/R	US	2011	173	52	18.1 (0.3)	0.3	Starting university	PA, Eating behaviours	Y (U)	8
Hootman, K. C., et al. (2018)^30^	N/R	US	2011	241	52	18.1 (0.3)	0.3	Starting university	PA, Diet	N	8
Kwan, M. Y., et al. (2012)^31^	NPHS	Canada	1994	640	49	13.4 (1.3)	2, 4, 6, 8, 10, 12	Leaving high schoolStarting university	PA	N	6
Li, K., et al. (2016)^33^	NEXT Plus	US	2010	463	55	n/r	1	Leaving high schoolStarting university	PA	Y (A)	8
Li, K., et al. (2016)^32^	NGHS	US	2010	2659	55	16.2 (SE = 0.02)	1	Leaving high schoolStarting university Starting employment	PA	N	7
Miller, J., et al. (2018)^36^	ProjectEAT	US	1998/9	4746	50	14.9 (1.7)	5, 10, 15	Starting university Leaving university Starting employment	PA	Y (U)	7
Molina‐Garcia, J., et al. (2015)^21^	N/R	Spain	2011	244	59	17.6 (0.7)	1	Leaving high school	PA	Y (A)	5
Paluch, A. E., et al. (2018)^35^	N/R	US	2011	404	52	27.8 (3.7)	0.25	Starting employment Leaving university	PA	N	7
Parker, P. D., et al. (2010)^44^	N/R	Australia	N/R	212	62	17 (0.96)	1	Leaving high school	PA	Y (A)	3
Pullman, A. W., et al. (2009)^15^	N/R	Canada	2006	108	0	18.5 (SE = 0.1)	0.33	Starting university	Body weight, PA, Diet	N	7
Simons, D., et al. (2015)^25^	Rural activity project (RAP)	Australia	2003	440	49	17.6 (0.6)	1	Leaving high schoolStarting university Starting employment	PA	Y (A)	5
Ullrich‐French, S., et al. (2013)^24^	N/R	US	N/R	236	70	18.7 (0.3)	0.5	Starting university	PA	Y (U)	6
Winpenny, E.M., et al. (2018)^9^	NLHBS	Norway	1991	1100	46	14	1, 2, 4, 5, 7, 9, 16	Leaving education (school or university) Starting employment	Diet	N	6

Abbreviations: ALSWH: Australian Longitudinal Study on Women's Health; N/R: not reported; NGHS: NEXT Generation Health Study; NLSY97: National Longitudinal Survey of Youth 1997; NPHS: National Population Health Survey; PA: physical activity; SD: standard deviation; y: years. Studies that were meta‐analysed are designated to which transition they contributed: A, leaving high school to all destinations; U, leaving high school and starting university.

a Range given where mean (SD) not reported.

Table 3 also presents the results of the quality assessment of the included papers. Of the 19 papers included, 10 achieved a score of 7 or higher, suggesting high methodological quality. Few papers presented data showing nonselective nonresponse during follow‐up measurement, only 6 papers included objectively measured weight (n=2) or physical activity data (n=2), or a comprehensive assessment of diet (n=3), and several papers (n=7) reported on small samples of <250 participants.

### 3.1 Leaving high school or starting university: changes in physical activity

There were 14 papers that reported on the transition out of high school or into university/college (Table 3), of which 13 papers included data on change in physical activity across this transition. One of these papers (Hootman et al 2018)^29^ was not included in the analysis since the PA data reported on the same sample was already included from another paper^30^; this paper had been retained in the review due to further information on change in diet. The remaining 12 papers reported studies which followed individuals recruited during high school to subsequent follow‐up after high school (n=8), studies which recruited individuals shortly before they entered university and followed‐up during university (n=3), and one study which recruited individuals from high school and followed‐up in a university setting (n=1). Those studies that followed individuals from high school to posthigh school varied in the extent to which they analysed the education or employment destinations of the individuals in the study. Details of the transitions that were assessed from each study are documented in Table 3, with an indication of how they were included in the meta‐analysis. We have grouped leaving high school and entering university transitions together in our analysis since these transitions were analysed together in many papers, and they typically occur at the same time in people's lives.

**Figure 2:**
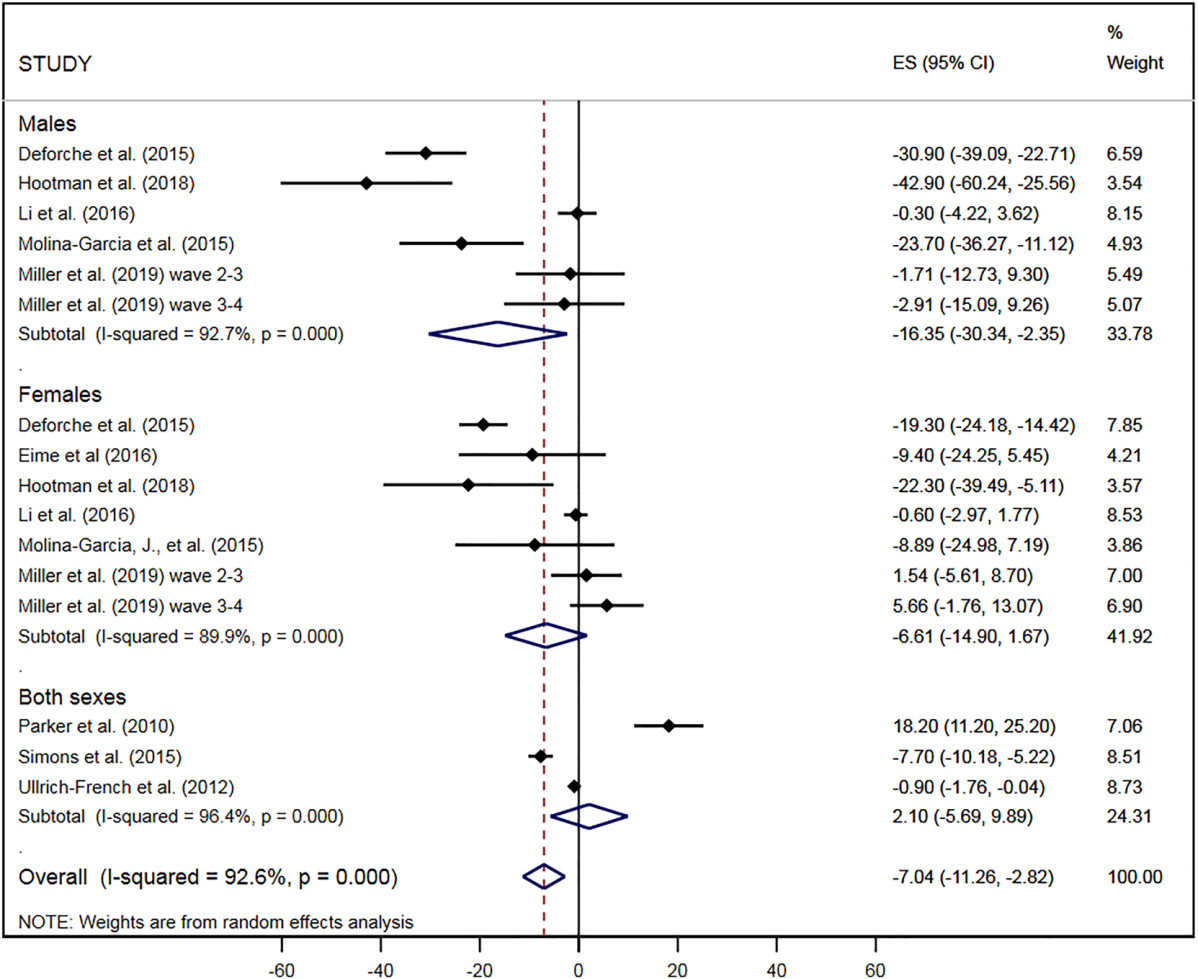
Change in moderate‐to‐vigorous physical activity (min/day) on leaving high school or starting university, from all eligible studies, stratified by sex

Nine studies reported data that could be converted to minutes/day of MVPA and included in a meta‐analysis of changes in PA as individuals leave high school (see Figure 2). Results of the meta‐analysis suggest that overall, leaving high school is associated with a decrease of ‐7.04 (95% CI, −11.26, −2.82) minutes/day of MVPA over an average follow‐up of 0.8 years. Stratified meta‐analysis showed that the decrease in MVPA was larger for males (−16.35 [−30.34, −2.35] min/day of MVPA) than for females (−6.61 [−14.90, 1.67] min/day of MVPA). Of the remaining three studies, which could not be included in the meta‐analysis, all reported declines in physical activity over the transition out of high school.^15^, ^31^, ^32^ Visual inspection of funnel plot asymmetry and Egger's test for bias suggested no evidence of asymmetry in the meta‐analysis (*P*=.84) and therefore no evidence of publication bias.

**Figure 3:**
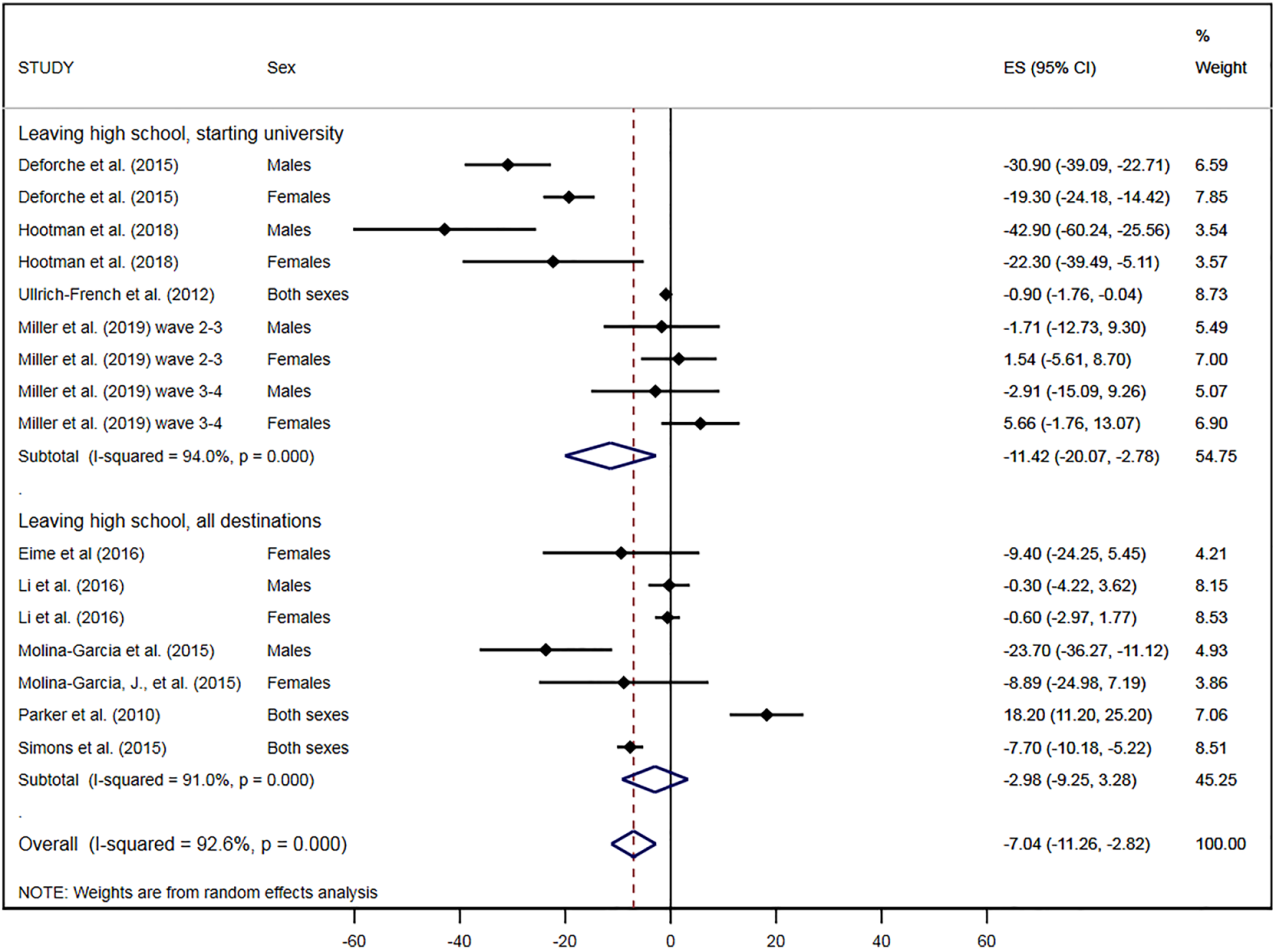
Change in moderate‐to‐vigorous physical activity (min/day) on leaving high school or starting university, from all eligible studies, stratified by type of transition

Since the studies included in this transition covered both leaving high school and starting university transitions, we performed stratified analysis separating the studies which presented data on changes in MVPA on leaving high school to all destinations (which could include participants transitioning to further study, employment, or other activities) from those that specified that changes were across the high school into university transition (Figure 3). This stratified meta‐analysis showed a larger effect for studies looking at the transition from high school to university (−11.42 (95% CI, −20.07, −2.78) min/day of MVPA), and no effect for studies focused on the transition from high school to any destination (−2.98 [95%CI, −9.25, 3.28) min/day of MVPA).

Two studies, which were included in the “leaving school, all destinations” subgroup of the meta‐analysis, conducted further analysis to take into account the destination of those leaving high school, although sufficient data were not available for these findings to be included as part of our meta‐analysis. These studies both reported that those continuing to college/university experienced a smaller decrease in MVPA compared with those not continuing in education.^25^, ^33^ However, a further study, which could not be included in the meta‐analysis due to its dichotomized outcome, reported higher odds of meeting PA recommendations among those not attending college/university following high school compared with those attending college/university.^32^

Two studies that investigated the transition of leaving high school, reporting an overall decrease in physical activity across the transition, included further analysis of changes in physical activity among subgroups who were in work compared with not in work at follow‐up. One study reported that those working full‐time or part‐time were more likely to meet PA recommendations in the year following high school, compared with those not working (including those in education).^32^ A second study reported no significant difference in decrease in physical activity between transitioning from high school into full‐time work vs not full‐time work.^25^

### 3.2 Leaving high school or starting university: Changes in body weight, diet, and eating behaviours

We identified three papers that reported on changes in body weight or adiposity, all of which addressed the transition of leaving high school and reported increases in weight across the transition from high school to university.^13^, ^15^, ^34^ Deforche et al reported an increase in weight of 4.2 kg (95% CI, 3.4, 5.0) for men and 1.9 kg (95% CI, 1.4, 2.5) for women over 1.5 years,^13^ while Pullman et al reported an increase in weight among men of 1.4 kg (*P* <.05) over 2 to 6 months.^15^ Baum et al reported gains in weight in both those who enter their first year of college/university and those of the same age who did not begin college/university, with an additional weight gain of 0.5 kg from age 16 to first year of college among those attending vs not attending college/university.^34^

Four papers reported on changes in diet or eating behaviours across the transition from high school to university. Deforche et al reported decreases in consumption of fruits and vegetables and dairy products, as well as a decrease in consumption of discretionary foods (soft drinks and snacks) across the transition,^13^ with no significant differences between males and females.^13^ Similarly, decreases in consumption of fruit and dairy products, but not vegetables, grains, meat, soft drinks, or coffee/tea, were seen in a sample of male Canadian students.^15^ Two papers reported results from one study in the United States, which found small decreases in meal and snack consumption^29^ and decreases in total energy intake^30^ across the high school to university transition.

### 3.3 Leaving university: Changes in physical activity and diet

Four studies investigated the association of leaving university with changes in physical activity. Paluch et al and Miller et al reported no significant change in physical activity associated with graduation from university.^35^, ^36^ Carney et al investigated physical activity across the transition of leaving university using a stages of change model. They reported that of those who were physically active during university, 22% were no longer physically active at recommended levels following leaving university, but no statistical test is reported.^37^ Bell et al found no significant change in physical activity associated with moving from further education to work.^38^

One study investigated changes in diet on leaving full‐time education.^9^ This study analysed leaving full‐time education at any age rather than specifically leaving university, but the study population included around 60% of individuals who continued in education beyond age 18. Leaving education, after adjustment for underlying trends and other life transitions, was associated with no changes in consumption of fruit and vegetables, but an increase in consumption of confectionery (0.33 times/week [95% CI, 0.04, 0.62]) and sugar‐sweetened beverages (0.49 times/week [95% CI, 0.10, 0.87]), a change which was larger in females than males.

### 3.4 Starting employment: Changes in physical activity and diet

Three papers examined transitions in employment status, separately from any consideration of education status. In a US population of mean age 28 years, Paluch et al reported that starting a first job was associated with a decrease of 18.7 minute/day of MVPA in both males and females (SD 9.0 males and SD 5.4 females), within a 3‐month period, while leaving a job was associated with an increase in MVPA of 12.7 (SD 4.2) minute/day in women and no significant change in men (−4.8 [SD 6.1]).^35^ Based on data from the Australian Longitudinal Study on Women's Health (ages 22‐27 at baseline), Brown et al reported that beginning work outside the home was associated with reduced odds of increasing physical activity and no difference in odds of decreasing PA, compared with a group who did not begin work over the same 3‐year period.^39^ Miller et al reported no significant changes in MVPA associated with starting employment.^36^

One study investigated the association of starting employment with changes in diet.^9^ After adjustment for underlying trends and other life transitions, Winpenny et al reported that entering employment was not associated with significant changes in frequency of consumption of fruit, vegetables, confectionery, or sugar‐sweetened beverages.

## 4 DISCUSSION

### 4.1 Main findings

The most studied transition among the education and employment transitions investigated in this literature review was the transition of leaving high school, with a large proportion of those studies reporting on the transition from high school to college or university. Our meta‐analysis of change in MVPA across this transition showed that MVPA decreased by around 7 minutes/day across this transition, with a greater decrease among males than females. Fewer papers reported on changes in diet and body weight across this transition, but overall the data suggested increases in body weight and decreases in diet quality from high school to university.

A very small number of studies looked at other education or employment transitions, including starting or leaving employment or leaving university. These suggested that leaving university was not associated with changes in physical activity and that starting employment (separately from leaving education) is associated with a decrease in activity. One study on diet reported that leaving university was associated with increases of consumption of confectionery and sugar‐sweetened beverages, but starting employment was not associated with changes in diet quality.

### 4.2 Relationship to prior knowledge

The evidence on changes in physical activity across the transition of leaving school is in line with other evidence that has suggested that physical activity decreases from adolescence into early adulthood by −5.2 (−7.3 to –3.1) minute/day MVPA over a mean of 3.4 years.^7^ Our finding of a decline of −7.0 (−14.8 to −4.8) minute/day of MVPA across the transition out of high school over a mean of 0.8 years suggests that this is an important transition beyond the underlying trend in decreasing PA over this age range. We found that the decrease in MVPA was larger in males (−16.35 [95% CI, −30.34, −2.35] min/day) than females (−6.61 [95% CI, −14.90, 1.67] min/day), and again, this is in line with previous evidence on declines from adolescence to early adulthood,^7^ which may be in part due to higher levels of physical activity in high school among boys compared with girls.

Our stratified meta‐analysis suggested a larger decrease in MVPA among those transitioning from high school to university than for populations leaving high school but not moving to a specified destination. However, this conclusion differed from that of two studies that analysed this question directly, which reported that those continuing to university experience a smaller decrease in physical activity compared with those not continuing in education.^25^, ^33^ The impact of such transitions will likely differ between different settings, due to differences in the home and institutional environments that individuals are moving from and to, and results will also depend upon the methods used to analyse such associations. We did not have sufficient studies in this review to conduct further analysis of these potential contributory factors; however, further research is warranted to generate a better understanding of the factors driving changes in physical activity among those leaving school, to different destinations and among different socioeconomic groups.

Our findings on changes in body weight across the transition from high school to university are in line with reviews that have focused on the “Freshman 15” weight gain within the first year of university, with all studies included in this review reporting an overall increase in body weight across this transition.^11^, ^12^ In this review, we focussed specifically on life transitions and therefore only included studies that assess changes from before to during university, resulting in a lower number of studies than found in “Freshman 15”‐based literature reviews, which focus on changes within the first year spent at university.^11^, ^12^ If changes in behaviour are associated with the changes in social and physical environment resulting from the move from the home/school to university environment, it is likely that diet and physical activity behaviours may change very quickly as individuals go through this transition, rather than in the following months. However, individuals may continue to gain weight following the shift to new behaviour patterns, suggesting that changes in body weight that begin at the time of transition may be expected to continue into the freshman year (first year of university). Studies of the “Freshman 15” phenomenon that have investigated the changes in behaviour driving this weight gain have suggested that changes in diet and physical activity are likely to contribute,^40^ although increases in alcohol consumption and psychological stress may also play a role.^11^ In this review, the four papers reporting data on changes in diet were hard to synthesize due to reporting of different measures; however, reporting of decreases in fruit and dairy consumption was consistent across two studies. The reported overall decreases in consumption and decreases in total energy intake in some of these papers may be due to misreporting or may reflect partial compensation for the reductions in physical activity across this transition.

### 4.3 Strengths and limitations

This literature review represents the first synthesis of evidence across the education and employment transitions from adolescence into early adulthood, allowing us to begin to understand the impact that such life transitions may have on lifestyle behaviours and adiposity. We only included studies that reported data from before and after a transition, meaning that studies reporting changes in behaviour during the period after beginning university or starting a new job would have been excluded. This allowed us to specifically focus on the changes across the life transitions themselves but resulted in a more restricted set of studies than other reviews that have focused on this age range.^7^, ^12^, ^41^

Our review identified consistent evidence of a decrease in physical activity and increase in body weight across the transitions out of high school and into university. Across other transitions and outcomes, our review was limited by the paucity of evidence,^7^, ^41^ and further primary studies are needed. Our meta‐analysis found a significant decrease in physical activity on leaving high school; however, the analysis showed a high degree of heterogeneity between studies. There were not enough studies available to test further explanatory variables using meta‐regression; however, it may be that differences in study population, in structure and culture of different higher education institutions, and differences in study quality and methodology such as physical activity measurement method and length of follow‐up contributed to this heterogeneity. While this heterogeneity is to some extent accounted for by use of random effects meta‐analysis, such a high degree of heterogeneity suggests that pooled results should be interpreted with caution. Indeed, there was considerable variation in methods of collection of physical activity data, with only one of the studies included in the meta‐analysis using an objective method of physical activity measurement, and conversions were required in some studies between the reported data and the common units used for meta‐analysis.

In this literature review, we conducted a very wide‐ranging search, including all longitudinal prospective quantitative studies, from any country. We did not restrict our review based on study quality, and while this allowed for inclusion of more studies, it means that our findings are limited by the quality of the included studies, with only 10 of 19 studies judged to be of high methodological quality, with, for example, most studies of physical activity relying on self‐reported measures of measurement (n=15/17). We did not observe differences in findings based on study quality, although the opportunity for such comparisons was limited by the small number of studies in each transition/outcome combination. Further research using adequately powered studies, which include objective measures of adiposity and physical activity and comprehensive measures of dietary intake over these transitions, is recommended. In addition, the studies found in this literature review were all situated in North America, Australia, or Europe, and so results may not be generalizable to other regions.

One of the challenges of understanding the impact of early adulthood transitions is the density of transitions that occurs during this life stage. Individuals may be going through many parallel changes for example changes in living arrangements, education and employment status, and relationships. Many of the papers included in this review focussed on a single transition and specified the before and after situation (eg, high school to university). However, other papers studied transitions that were less well specified (eg, leaving high school or leaving university), with no analysis of the destination to which participants were moving. In such cases, it will be important to understand whether it was really the effect of, eg, “leaving high school,” which was responsible for a change in behaviour or whether it was the destination following the transition. Several studies in this review reported on more than one transition,^25^, ^39^ which might provide an opportunity to investigate how transitions might combine to influence changes in outcome; however, most, with only a few exceptions (eg, Li et al^33^ and Winpenny et al^9^), analysed transitions independently and did not cross‐control for the other transitions reported. Similarly, many of the studies included in this review did not take account of any underlying changes in behaviour, which may be independent of the transition, for example, an underlying trend of decreasing physical activity from adolescence to adulthood.^7^ In order to understand the factors driving changes in behaviour in early adulthood, there is a need for more sophisticated longitudinal analyses, which superimpose analysis of specific transitions on underlying trends in behaviour and adjust for other transitions which may cooccur and be associated with the outcome of interest (see, for example, Winpenny et al.^9^).

### 4.4 Implications for policy, practice, and research

The findings from this review, together with evidence of early adulthood as a time for increasing overweight/obesity prevalence^6^ and decreasing PA,^7^ suggest that there is a need to focus on the transition of leaving high school as a target for obesity prevention. While there has been considerable focus on school‐based public health policy and interventions, there has been much less interest on how exposures may change as individuals leave school and enter further education or employment. It has been suggested that school is a protective environment for physical activity maintenance, with decreases in physical activity in adolescence occurring more during weekend days than week days.^23^ Limited evidence has suggested that university eating environments may influence eating behaviours and diet,^42^ while physical environment and university characteristics contribute to university students' physical activity levels.^43^ More work is needed to understand how policies and interventions might be developed to continue to support individuals as they move from the school environment, into higher education environments and into the workplace.

## 5 CONCLUSION

In this review, we have found consistent evidence of a decrease in physical activity and an increase in body weight across the transitions out of high school and into university, suggesting a need for policies and interventions to support individuals as they transition out of high school. There is little consistent evidence on changes in diet and little evidence available on transitions among those not entering further education, and across transitions of leaving university and starting employment. High quality longitudinal studies are needed to understand the combinations of life transitions, which may be associated with deterioration of lifestyle behaviours and gains in adiposity as individuals enter adulthood. In addition, further detailed analysis is needed to understand the determinants of changes in behaviour across these transitions and to provide evidence on which to base development of new public health policies and interventions in these populations.

## FUNDING

This study was supported by the Centre for Diet and Activity Research (CEDAR), a UKCRC Public Health Research Centre of Excellence (RES‐590‐28‐0002). Funding from the British Heart Foundation, Department of Health, Economic and Social Research Council, Medical Research Council, and the Wellcome Trust, under the auspices of the UK Clinical Research Collaboration, is gratefully acknowledged. The work is additionally supported by the Medical Research Council (MC_UU_12015/7). Rebecca Love is funded by a Gates Cambridge Scholarship. Campbell Foubister is funded by a NIHR School for Public Health PhD Studentship. 

## CONFLICTS OF INTEREST

No conflict of interest was declared.

## Supporting information

Table S1: Search terms.
Table S2: Quality assessment tool for longitudinal observational studies of diet.
