# Supplementary material for: Changes in physical activity, diet, and body weight across the education and employment transitions of early adulthood: A systematic review and meta‐analysis
Source: Obes Rev. 2020 Jan 19;21(4):e12962. doi: 10.1111/obr.12962 (PMC7079102; doi:10.1111/obr.12962)
Supplement: Supplementary file 1 — Table S1: Search terms. Table S2: Quality assessment tool for longitudinal observational studies of diet. [file OBR-21-e12962-s001.pdf]

SUPPORTING INFORMATION

Changes in physical activity, diet and body weight across the education and employment transitions of early adulthood: A systematic review and meta-analysis.

Eleanor M. Winpenny, Miranda Smith, Tarra Penney, Campbell Foubister, Justin M. Guagliano, Rebecca Love, Chloe Clifford Astbury, Esther M. F. van Sluijs, Kirsten Corder.

Address for correspondence: Eleanor Winpenny, MRC Epidemiology Unit, University of Cambridge School of Clinical Medicine, Box 285, Institute of Metabolic Science, Cambridge Biomedical Campus, Cambridge, CB2 0QQ, U.K. Telephone: +44 (0)1223 769150 Fax: +44 (0)1223 330316 Email: ew470@cam.ac.uk

Table S1: Search terms.

1	Diet outcomes	food*[Title/Abstract] OR beverage*[Title/Abstract] OR nutrient*[Title/Abstract] OR macronutrient*[Title/Abstract] OR “energy intake”[Title/Abstract] OR diet[Title/Abstract] OR diets[Title/Abstract] OR “dietary”[Title/Abstract] OR nutrition[Title/Abstract] OR nutritional[Title/Abstract] OR fruit[Title/Abstract] OR vegetable[Title/Abstract] OR fruits[Title/Abstract] OR vegetables[Title/Abstract] OR dairy[Title/Abstract] OR grain*[Title/Abstract] OR meat[Title/Abstract] OR cereal*[Title/Abstract] OR “soft drink*”[Title/Abstract] OR soda[Title/Abstract] OR SSB[Title/Abstract] OR SSBs[Title/Abstract] OR salt[Title/Abstract] OR sugar*[Title/Abstract] OR "Food"[Mesh] OR "Beverages"[Mesh] OR diet[Mesh] OR "Nutrition Surveys"[Mesh] OR "Diet Records"[Mesh] OR "Dietary Fats"[Mesh] OR "Dietary Proteins"[Mesh] OR "Dietary Carbohydrates "[Mesh] OR "Healthy Diet"[Mesh]
	Eating Behaviour outcomes	OR cook*[Title/Abstract] OR “food preparation”[Title/Abstract] OR “eating behaviour”[Title/Abstract] OR “eating behavior”[Title/Abstract] OR meal*[Title/Abstract] OR snack*[Title/Abstract] OR “dining out”[Title/Abstract] OR breakfast[Title/Abstract] OR dinner[Title/Abstract] OR lunch[Title/Abstract] OR supper[Title/Abstract] OR “fast food”[Title/Abstract] OR “fast-food”[Title/Abstract] OR restaurant[Title/Abstract] OR “take-away”[Title/Abstract] OR takeaway[Title/Abstract] OR “eating out”[Title/Abstract] OR “away from home”[Title/Abstract] OR “out of home”[Title/Abstract] OR “away-from-home”[Title/Abstract] OR “out-of-home”[Title/Abstract] OR “home-cook*”[Title/Abstract] OR “home prepar*”[Title/Abstract] OR “home cook*”[Title/Abstract] OR “home-prepar*”[Title/Abstract]
	Physical activity outcomes	OR "physical activity"[Title/Abstract] OR "physical activities"[Title/Abstract] OR “physically active”[Title/Abstract] OR "active transport"[Title/Abstract] OR "active travel"[Title/Abstract] OR exercise*[Title/Abstract] OR cycle[Title/Abstract] OR cycling[Title/Abstract] OR walk*[Title/Abstract] OR sport*[Title/Abstract] OR "energy expenditure"[Title/Abstract] OR MVPA[Title/Abstract] OR “vigorous activity”[Title/Abstract] OR VPA[Title/Abstract] OR “moderate activity” [Title/Abstract] OR “light activity”[Title/Abstract] OR MPA[Title/Abstract] OR “intensity activity”[Title/Abstract] OR “LPA”[Title/Abstract] OR “strenuous activity”[Title/Abstract] OR “Exercise”[MeSH] OR “Sports”[Mesh]

	Adiposity outcomes	OR “BMI”[Title/Abstract] OR "weight gain"[Title/Abstract] OR "weight loss"[Title/Abstract] OR "body weight"[Title/Abstract] OR "body mass"[Title/Abstract] OR "change in weight"[Title/Abstract] OR " weight change"[Title/Abstract] OR "waist circumference"[Title/Abstract] OR anthropometry[Title/Abstract] OR anthropometric[Title/Abstract] OR “fat mass”[Title/Abstract] OR “lean mass”[Title/Abstract] OR “body composition”[Title/Abstract] OR skinfold[Title/Abstract] OR overweight[Title/Abstract] OR obesity[Title/Abstract] OR adiposity[Title/Abstract]
2	Longitudinal	longitudinal[Title/Abstract] OR cohort[Title/Abstract] OR prospective[Title/Abstract] OR “follow-up*”[Title/Abstract] OR "follow up"[Title/Abstract] OR "Follow-Up Studies"[Mesh] OR “Prospective Studies”[Mesh] OR “Longitudinal Studies”[Mesh] OR “Cohort Studies”[Mesh] OR “life-course”[Title/Abstract] OR “life course”[Title/Abstract] OR “repeated measure”[Title/Abstract] OR “repeated-measure”[Title/Abstract]
3	Transition	“Further education”[Title/Abstract] OR “Higher education”[Title/Abstract] OR “entering education”[Title/Abstract] OR “leaving education”[Title/Abstract] OR “leaving school”[Title/Abstract] OR “school leaver*”[Title/Abstract] OR college[Title/Abstract] OR university[Title/Abstract] OR freshman[Title/Abstract] OR freshmen[Title/Abstract] OR Graduate[Title/Abstract] OR Undergrad*[Title/Abstract] OR employment[Title/Abstract] OR “first job”[Title/Abstract] OR “starting work” [Title/Abstract] OR “moving house”[Title/Abstract] OR “change of address”[Title/Abstract] OR “residential relocation”[Title/Abstract] OR “residential mobility”[Title/Abstract] OR “residential instability”[Title/Abstract] OR “geographic* mobility”[Title/Abstract] OR “geographic relocation”[Title/Abstract] OR “residential relocation”[Title/Abstract] OR “residential stability”[Title/Abstract] OR<br><br>OR “living arrangements”[Title/Abstract] OR cohabit*[Title/Abstract] OR marriage[Title/Abstract] OR “marital status” [Title/Abstract] OR parity[Title/Abstract] OR parous[Title/Abstract] OR childbearing[Title/Abstract] OR postpartum[Title/Abstract] OR “post-partum”[Title/Abstract] OR "first child*"[Title/Abstract] OR "having children"[Title/Abstract] OR "having a child"[Title/Abstract] OR trajector*[Title/Abstract] OR “transition*”[Title/Abstract] OR “life transition”[Title/Abstract] OR “life events”[Title/Abstract] OR “Life Change Events"[Mesh] OR "Marital Status"[Mesh] OR "Employment"[Mesh]
4	Additional filters	English[lang]<br><br>NOT Review[ptyp]<br><br>NOT Neoplasm[Mesh]<br><br>Humans (filter)
5		1 AND 2 AND 3 AND 4

Note. The search strategy was originally designed for PubMed and then adapted as necessary for the other databases.

Table S2: Quality assessment tool for longitudinal observational studies of diet.

Item	Physical activity/sedentary behaviour	Diet	Anthropometry (Body weight, BMI, waist circumference)
A. Study population and participation (baseline): the study sample represents the population of interest on key characteristics.			
1. Adequate description of sampling frame, recruitment methods, period of recruitment and place of recruitment	Item was scored positively if three of the following points were mentioned: (i) description of how participants were sampled (e.g. sourced from the electoral roll, all schools in state or country, or from a larger study); (ii) description of specific methods used for recruitment (e.g. newsletters, phone call, advertising); (iii) period of recruitment provided (e.g. March 2010); or (iv) place of recruitment detailed (e.g. Wollongong, Australia, Glasgow)		
2. Adequate description of baseline study sample for key characteristics	Item was scored positively if all three of the following points were mentioned: (i) number of participants; (ii) age (mean age or % at each age); and (iii) gender		
B. Study attrition: loss to follow-up not associated with key characteristics (i.e. the study data adequately represent the sample)			
3. Provision of the exact number of participants at each follow-up measurement(s)	Item was scored positively if the number or percentage of participants at each time point was detailed		
4. Provision of exact information on follow-up duration	Item was scored positively if detail about the follow-up duration (e.g. 1 year, 6 years) was provided		
5. Presentation of data showing non-selective non-response during follow-up measurement(s)	Item was scored positively if those who dropped out of the study were similar on key characteristics to those who were retained at follow-up.		
C. Data collection			
6. Adequate description of methods of data collection	Objective measures: Item was scored positively if at least three of the following points were mentioned: (1) type of instrument; (2) length of epoch; (3) number of days worn; (4) number of hours/day worn; (5) number of	Comprehensive measurement of diet: item was scored positively if both of the following points were mentioned: (i) type of instrument; (ii) number of days measured.	Objective measurement: item was scored positively if both of the following points were mentioned: (i) type of instrument; (ii) who performed the measurements.

	minutes monitored; (6) description of monitor placement; and (7) data reduction methods described		
	Subjective measures: Item was scored positively if the instrument was described including the number of items.	Questionnaire measures: Item was scored *positively* if the questions used and response options were described in full.	Self-reported: N/A
7. Adequate measurement method	Item was scored positively if physical activity was measured objectively and/or sedentary behaviour was measured by proxy report, including provision of validity and/or reliability data and a reference	Item was scored positively if a comprehensive diet measurement instrument (e.g. diet record, 24-hr recall, FFQ) was used. 0 points scored for any other method e.g. questions in a questionnaire	Item was scored positively if objective measurement, 0 if self-reported
8. Data processing	Item was scored positively if the cut points were referenced (subjective measures were given an N/A, which meant that those studies were scored using a total of 9 rather than 10)	Item was scored positively if a description of adjustment for mis-reporting was included.	N/A
D. Data analyses			
9. Adequate description of analysed sample (inclusion and exclusion criteria)	Item was scored positively if details of the samples included in the final analysis were included (e.g. all participants; participants with complete data)		
10. The analysed sample was at least medium in size	Item was scored *positively* if analysed sample was ≥250 participants		

When multiple outcomes were assessed by a single paper, a positive score was given if any of the outcomes scored positively.
Scores for each item were summed and a paper was deemed to have high methodologic quality if it scored ≥7.
